# Quality of care for chronic conditions: identifying specificities of quality aims based on scoping review and Delphi survey

**DOI:** 10.1080/16549716.2024.2381878

**Published:** 2024-08-16

**Authors:** Grace Marie V. Ku, Willem van de Put, Deogratias Katsuva, Mohamad Ali Ag Ahmed, Megumi Rosenberg, Bruno Meessen

**Affiliations:** a Department of Public Health, Institute of Tropical Medicine, Antwerp, Belgium; b Department of Gerontology, Faculty of Medicine & Pharmacy, Vrije Universiteit Brussel, Brussels, Belgium; c Department of Preventive Medicine, Faculty of Medicine & Surgery, University of Santo Tomas, Manila, Philippines; d Sherpa University Institute, Montreal, Canada; e Department of Health Management, Evaluation and Policy, School of Public Health, University of Montreal, Montreal, Canada; f Centre for Health Development, World Health Organization, Kobe, Japan; g Health Financing and Economics Department, World Health Organization, Geneva, Switzerland

**Keywords:** Quality of Care for Chronic Conditions, Chronic conditions, quality of care, chronic care quality aims, low- and middle-income countries, chronic care quality definition

## Abstract

There is a growing need to implement high quality chronic care to address the global burden of chronic conditions. However, to our knowledge, there have been no systematic attempts to define and specify aims for chronic care quality. To address this gap, we conducted a scoping review and Delphi survey to establish and validate comprehensive specifications. The Institute of Medicine’s (IOM) quality of care definition and aims were used as the foundation. We purposively selected articles from the scientific (n=48) and grey literature (n=26). We sought papers that acknowledged and unpacked the plurality of quality in chronic care and proposed or utilised frameworks, studied their implementation, or investigated at least two IOM quality care aims and implementation. Articles were analysed both deductively and inductively. The findings were validated through a Delphi survey involving 49 international chronic care experts with varied knowledge of, and experience in, low-and-middle-income countries. Considering the natural history of chronic conditions and the journey of a person with a chronic condition, we defined and identified the aims of chronic care quality. The six IOM aims apply with specific meanings. We identified a seventh aim, continuity, which relates to the issue of chronicity. The group endorsed our specifications and several participants gave contextualised interpretations and concrete examples. Chronic conditions pose specific challenges underscoring the relevance of tailoring quality of care aims. The next steps require a tailored definition and specific aims to improve, measure and assure the quality of chronic care.

## Paper context

- **Main findings**: While previously defined aims of good-quality care may also apply to chronic care quality, the nature of chronic conditions and ensuing healthcare needs warrant specifications for good-quality chronic care.
- **Added knowledge**: Our proposed definition and specific aims are tailored to the natural history of chronic conditions, and can serve as a guide on determining what can be deemed as good-quality chronic care.
- **Global health impact for policy and action**: This work, developed to guide further work on designing purchasing instruments to improve quality of chronic care, particularly in low- and middle-income countries, may also be a source of inspiration for other interventions aiming at improving quality of chronic care.

## Background

Chronic conditions, whether non-communicable or infectious, are broadly defined to last 1 year or more and require ongoing medical attention and/or limit activities of daily living [1]. These raise particular issues in terms of quality of care: persons with chronic conditions (PwCC) should be offered a seamless journey through time in the healthcare system, across services, providers, levels of care, etc. [2–5]. More fundamentally, although healthcare providers (HCP) are expected to deliver healthcare, having a chronic condition means that PwCC are in-charge of their own health on a day-to-day basis. Daily decisions they make have a huge impact on their own health outcomes and quality of life [6]. Thus, chronic care should include disease prevention and medication prescription activities; give focus on disability limitation, rehabilitation [7], and palliative care; and involve, enable and engage the PwCC (and their families) in taking care of the condition, controlling risks and promoting well-being (self-care) [8] considering not only biomedical but also psychosocial aspects *to adapt* and self-manage in the face of social, physical, and emotional challenges [9,10]. It is likewise crucial to acknowledge the reality of multimorbidity.

Although the above are delineated in standards of care for specific chronic conditions and are recognised in different models of chronic care, we did not find any established ready-to-use definition of what would be considered good-quality chronic care [11]. Also, while there has been considerable work around a ‘global’ definition of quality of care and its core ‘aims’ over the last decades [12–15], there has been no systematic attempts made yet, to the best of our knowledge, to specify quality aims for chronic care. Having a tailored definition would empower the many and various actors – policy-makers, health financiers/purchasing agencies, healthcare/service providers, healthcare regulators, accreditation agencies, researchers, trainers, PwCC themselves, etc. – who are committed to achieve ‘good quality chronic care’ and/or have the mandate to implement specific quality-enhancing interventions for chronic care.

The main objective of this study was to produce comprehensive specifications of aims of quality of care for chronic conditions.

### Conceptual issues

As a starting point, we adopted the definition of quality of care put forward by the Institute of Medicine (IOM) [13–15]: *Quality of care is the degree to which health services for individuals and populations increase the likelihood of desired health outcomes and are consistent with current professional knowledge*, noting that this generic definition needs to be contextualised and the outcomes to be determined.

While we are aware that different documents/reports have extended or reorganised these, we likewise took the IOM’s six aims as our base: effectiveness; efficiency; safety; equity; accessibility, timeliness, affordability; and person-centredness.

With the ideal that quality improvement should focus on the results that matter most to various actors (patients and their families, healthcare providers, regulators, decision makers, etc.), we defined ‘aim’ as any broad category of importance with intrinsic value, as a desired final outcome that is achieved to denote that care is of good quality.

## Methodology

This paper is one of the outputs of a larger study commissioned by the World Health Organization (WHO). The request was to produce a comprehensive conceptualisation of ‘quality health services for chronic conditions’ that can be used by actors considering interventions to improve health services for chronic conditions, in this case, purchasing arrangements as an instrument for improvement, with a particular attention to policy needs of low- and middle-income countries (LMICs). Here, we concentrate on the specifications of chronic care quality aims.

We reviewed relevant literature and convened international stakeholders of chronic care and quality in a Delphi survey.

We conducted a scoping review following the PRISMA extension guidelines [16] to systematically identify available information on quality of care for chronic conditions, identifying key concepts. We selected works that have acknowledged and unpacked the plurality of quality in chronic care, and which proposed/made use of frameworks or looked into two or more IOM aims of care quality, and studied or demonstrated implementation. The scoping review protocol is available from https://www.itg.be/en/research/research-themes/quality-of-care-for-chronic-conditions.

### Scientific publications

On 2 February 2022, search for scientific publications was conducted in the PubMed and Science Direct databases using specific search terms: *‘chronic condition’/’chronic illness’/’chronic disease’; ‘quality of healthcare’; ‘innovative care for chronic conditions’; ‘chronic care model’; ‘quality criteria’; ‘quality indicators’*; specific chronic conditions considered among top drivers of chronic disease burden [17] (*‘ischaemic heart disease’, ‘hypertension’ and ‘stroke’; ‘diabetes mellitus’; ‘chronic kidney disease’; ‘lung cancer’; ‘HIV/AIDS’; ‘chronic obstructive pulmonary disease’ and ‘bronchial asthma’*) and additional conditions as suggested by the WHO team *(‘chronic musculoskeletal conditions’; ‘chronic skin disease’*); and criteria: *written in English or French; publication years 2002–2021; among humans*.

### Other literature and documents

Search for grey/other literature (policies, circulars, publications not available from scientific search engines) was conducted using similar keywords but including general quality of care documents, and with broader year limitations (1999–2022) in the Google search engine. Additionally, contacts from the WHO and healthcare regulatory agencies, organisations with chronic disease programmes/projects, and various Ministries of Health and/or connected agencies were requested to share any documents they have produced as related to quality of care, specifically for chronic conditions.

Inasmuch as our preliminary searches in the two scientific data bases already had considerable yield (>7,000 each) and wherein ± 40% were duplicates, and that we were able to retrieve rich grey documents, a decision was made to no longer add more data bases to the minimum requisite for scoping reviews.

### Literature sifting

Scientific publications were sifted through Rayyan (www.rayyan.ai). This was done systematically by a minimum of two members of the research team. A third researcher resolved any disagreements amongst the two, as needed. Scientific publications were initially screened through the titles. Abstracts of the chosen documents were individually reviewed. Full articles were scrutinised and selected; only documents that are relevant to this study were included in the final selection.

Grey literature and other documents were purposively collected.

### Data extraction and analysis

Data retrieval was systematically initiated by at least one of the members of the research team and verified by a different member. We critically analysed the literature. We made use of deductive approaches based on the IOM quality aims, and inductive approaches to identify any additional quality aims; utilising our definition of ‘aim’ to guide both. Narrative synthesis of retrieved information was done. We brought forward concepts related to the aims of good-quality chronic care. We note that quality aims were not always explicitly stated. We then deduced the aims based on our critical analysis of the text using the IOM aims as our base. We also identified any quality aim not included in the IOM proposition. Furthermore, the analysis was reflective and iterative, going back to the literature as we identified additional concepts.

### Delphi survey

We invited 52 respondents representing various stakeholders of chronic care and quality (including PwCC and their carers) from all over the world. We prepared a list of ‘mid’- to ‘advanced’ level chronic care experts, gleaned through our own professional networks, references from colleagues or other known experts, relevant publications in peer-reviewed journals or the websites of relevant organisations, and from recommendations by different organisations (e.g. NCD Alliance, Global Alliance on Chronic Diseases), and supplemented by the WHO Team. A concern was to secure, to the extent possible, representation across genders, types of expertise, and settings of activities/experience (with focus on low- and middle- over high-income settings), covering the six WHO regions. We conducted two rounds of the Delphi survey via an online application, Mesydel (https://mesydel.com/en). The first step was to arrive at an agreement over our scoping review findings that build towards the chronic care quality framework, and to propose financing mechanisms to improve the quality of chronic care. Based on first-round results, the second round was conducted to fine-tune purchasing arrangements. Findings contributing to the chronic care quality framework beyond the definition and aims of quality chronic care and including those on financing mechanisms will be presented in separate papers. For this paper, we concentrated on Round 1 results relating to our proposed chronic care quality aims. The respondents provided a rich justification for why specific chronic care quality aims should be included in the free-flow component of the survey. We synthesised and critically analysed the responses, reflecting on our scoping review findings and contrasting and comparing all information collected.

## Results

**Figure 1.**
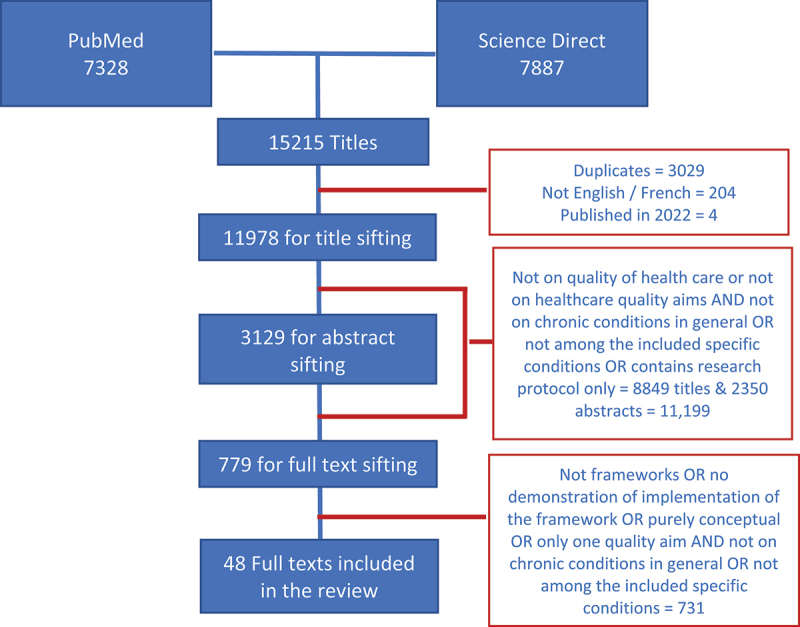
Sifting of retrieved scientific literature.

We retrieved 15,215 scientific articles and retained 48 [18–65] (Figure 1). The study designs were as follows: 17 reviews; 12 implementation research (quality improvement and/or model implementation); nine cross-sectional/surveys; three randomised controlled trials; three qualitative and mixed methods; two case studies; one qualitative study; and one position statement. Eighteen are specific for certain chronic conditions (diabetes = 5, cardiovascular diseases including hypertension and stroke = 5; HIV/AIDS = 2; chronic obstructive pulmonary disease = 2; chronic kidney disease = 2; osteoarthritis = 1; and cancer = 1). Some targeted specific groups (elderly = 5, children = 1, female = 1, informal caregiver = 1). Forty-six propose and implement or demonstrate implementations of various models of quality of care, mostly in high-income countries (*n* = 31), five in LMICs; South Africa = 3, Haiti = 1, not specified = 1), and the rest (*n* = 10) said to be global/international. Majority (*n* = 46) fit and consolidate the IOM definition of quality and two or more of the IOM care quality aims. A couple [46,47] consider Donabedian’s [66] elements of quality in healthcare.

* the ‘snowball’ search did not yield additional literature

We retrieved 26 grey literature/documents from the WHO (*n* = 10) [3,4,12,67–73]; the EU Joint Action on Chronic Diseases and Healthy Ageing Across the Life Cycle (*n* = 4) [74–77]; the IOM (*n* = 3) [14,15,78]; the United States of America (USA) Agency for Health Care Research & Quality (*n* = 2) [79,80]; and the rest coming from different agencies: two from the USA [81,82], and one document each from Australia [83], Belgium [84], Canada [85], Ireland [86], and the Philippines [87].

During data extraction, we noted that the scientific papers usually concentrate on a particular stage in what we deem to be the ‘journey’ of a person through healthcare, considering the natural history of (most) chronic conditions (e.g. risk-prevention/-control, follow-up, rehabilitation, etc.). We thus went back to our selection to consciously extract additional information that would expound on the relevance of various stages in a continuum of care relevant to the identified aims. We will discuss the concept of the PwCC journey in a related paper.

Tables 1 and 2 provide overviews of the scientific articles and grey literature and chronic care quality aims identified.

**Table 1.** Overview of the scientific articles and chronic care quality aims identified.

First author (Year)	Design	Country/ies of focus/study site	Framework used/proposed	Chronic condition of focus	Stage in the PwCC Journey	Quality aims identified (deductively identified)
Hung et al. (2007) [18]	Implementation research	USA	Chronic Care Model	Non-specific	At high risk (risk control)	Effectiveness Person-centredness
Lewanczuk et al. (2006) [19]	Literature review	Canada		Hypertension	Diagnosis	Effectiveness Continuity
Hung et al. (2008) [20]	Implementation research	USA		Non-specific	Follow-up (not in good control of the condition)	Effectiveness Person-centredness Continuity
Hroscikoski et al. (2006) [21]	Qualitative comparative case study	USA		Non-specific	Follow-up (general)	Effectiveness Person-centredness Continuity
Janssen et al. (2015) [22]	Cross-sectional/survey	Netherlands		Non-specific		Effectiveness Person-centredness
Kaissi et al. (2006) [23]	Cross-sectional/survey	USA		Diabetes		Effectiveness (Equity) Person-(and family-) centredness Continuity
Lim et al. (2018) [24]	Systematic review	Multiple		Diabetes	Follow-up (not in good control of the condition)	Effectiveness Person-centredness
Ludt et al. (2012) [25]	Cross-sectional/survey	Austria, Germany, Netherlands, Switzerland, and UK		Cardiovascular disease (CVD)	Follow-up (general)	Effectiveness Person-centredness
Lyon et al. (2011) [26]	Implementation research	USA		Non-specific		Effectiveness Efficiency Person centeredness
Vrijhoef et al. (2009) [27]	Qualitative & mixed methods	Netherlands		Non-specific		Effectiveness (Accessibility), Timeliness Person centeredness Continuity
Petrelli et al. (2021) [28]	Literature review	Italian context	Chronic care model	Non-specific	Follow-up (general)	Effectiveness Efficiency Person-centeredness Continuity
Lall et al. (2018) [29]	Qualitative methods	LMIC context		Non-specific		Effectiveness Equity Safety Accessibility, Timeliness Person-centeredness Continuity
Mateo et al. (2019) [30]	Implementation research	Spain		Diabetes		Effectiveness Efficiency
Adams & Wood (2016) [31]	Literature review	USA?		Non-specific Paediatric population		Effectiveness Accessibility Person-centeredness Continuity
Enderlin et al. (2013) [32]	Literature review	USA		Non-specific Older adults		Effectiveness Efficiency Person-centeredness Continuity
Sendall et al. (2016) [33]	Literature review	no focus on a specific country		Multi-morbidity Older adults	Follow-up (Multimorbid conditions)	Effectiveness Person-centredness Continuity
Hopman et al. (2016) [34]	Systematic review	no focus on a specific country				Effectiveness Person-centredness
Parchman & Kaissi (2009) [35]	Cross-sectional/survey	USA		Diabetes	Complications	Effectiveness Person-centredness
Litzelmann et al. (2019) [36]	Qualitative and mixed methods	USA		Cancer Informal caregiver	Informal caregiver	Effectiveness Person- (and caregiver-) centredness
Dugoff et al. (2013) [37]	Literature review	USA	Care coordination measures framework	Multi-morbidity	Follow-up (multi-morbidity)	Effectiveness Patient-centredness Continuity
Brand et al. (2014) [38]	Systematic review	no focus on a specific country	Chronic Disease Management model	Osteoarthritis	Follow-up	Effectiveness Person-centredness
Buja et al. (2018) [39]	Literature review (Umbrella review)	no focus on a specific country	Clinical governance framework	Non-specific		Effectiveness Efficiency Safety Person-centredness
Belland & Hollander (2011) [40]	Literature review	no focus on a specific country	Community-based model	Non-specific Older adults		Effectiveness Equity (Accessibility) Continuity
Kanter et al. (2013) [41]	Implementation research	USA	Complete Care Model	Non-specific	All steps	Effectiveness Person-centredness Continuity
Chiu et al. (2020) [42]	Implementation research	Canada	End-of-life	Chronic kidney disease (CKD)	End of life	Effectiveness Person-centredness Continuity
Morrin et al. (2013) [43]	Implementation research	Canada	Healthy Living Program	Diabetes	Prevention Follow-up (multi-morbidity)	Effectiveness Efficiency Equity Person-centredness
Nuno et al. (2012) [44]	Literature review	no focus on a specific country	Innovative Care for Chronic Conditions Model	Non-specific	All steps	Effectiveness Efficiency Person-centredness Continuity
Lebina et al. (2020) [45]	Qualitative and mixed methods	South Africa	Integrated Chronic Disease Care (Note: Ameh et al., 2017A & 2017B make use of Donabedian’s Quality Framework)	Non-specific	Follow-up (general; co-morbidities?)	Efficiency Accessibility
Ameh et al. (2017) [46]	Cross-sectional survey	South Africa		Non-specific		Efficiency Accessibility Continuity
Ameh et al. (2017) [47]	Case study	South Africa		Non-specific		Efficiency Accessibility
Ulbrich et al. (2017) [48]	Literature review (integrative review)	no focus on a specific country	Multiple models	Non-specific	Follow-up (general)	Efficiency Person-centredness
Grover & Joshi (2015) [49]	Systematic review	Majority of the studies US-based		Multiple chronic conditions (diabetes, CVD, Chronic obstructive pulmonary disease | COPD)	Prevention & Follow-up	Effectiveness, including cultural effectiveness Efficiency Person- (and family-) centredness Continuity
Disler et al. (2012) [50]	Literature review (Integrative review)	no focus on a specific country	Palliative care	COPD	Palliative/End of life	Effectiveness Accessibility Person-centredness
Kari et al. (2021) [51]	Randomised Controlled Trial (RCT)	FInland	People-centred care model	Non-specific Older adults	Follow-up	Effectiveness Efficiency Person-centredness
Kamajian et al. (2010) [52]	Implementation research	USA	People-centred medical home model	Non-specific Older adults		Effectiveness Efficiency Safety Person-centredness
Brownson et al. (2007) [53]	Cross-sectional/survey	USA	(continuous) quality improvement model (plan-do-check-act)	Non-specific	Follow-up (general)	Effectiveness Person-centredness
Harvey et al. (2015) [54]	Quasi-experimental	United Kingdom		CKD	Diagnosis & Follow up	Effectiveness Efficiency
Hayashino et al. (2015) [55]	cluster RCT	Japan		Diabetes	Follow-up (regular)	Effectiveness Efficiency
Hirschhorn et al. (2009) [56]	Implementation research	USA		HIV/AIDS		Accessibility Continuity
Joseph et al. (2015) [57]	Implementation research	Haiti		HIV/AIDS		Effectiveness Efficiency Accessibility Continuity
Pullen et al. (2021) [58]	RCT	UK and USA	(continuous) quality improvement model (plan-do-check-act)	COPD	Diagnosis & Follow-up	Effectiveness Accessibility Person-centredness
Wellwood et al. (2011) [59]	Systematic review	France, Italy, Sweden, Spain, Lithuania, Poland, United Kingdom		Stroke	Complications	Effectiveness Accessibility Continuity
Hawthorne et al. (2012) [60]	Cross-sectional/survey	the United Kingdom	Quality of outcomes framework	Diabetes	Follow-up (regular)	Effectiveness Person-centredness
Fletcher et al. (2012) [61]	Literature review	USA	Team based care model	CVD	Follow-up (general)	Effectiveness Efficiency Continuity
Mitchell et al. (2019) [62]	Cross-sectional/survey	USA		Non-specific		Effectiveness Efficiency (person-centredness)
Van Houtven et al. (2019) [63]	Implementation research	USA		Non-specific		Effectiveness Person- and family- centredness Continuity
Washington et al. (2011) [64]	Cross-sectional/survey	USA	Women’s primary care model	Non-specific Women	Follow-up (women)	(Effectiveness) Equity Person-centredness
Campbell et al. (2012) [65]	Position statement	Canada	Unspecified	Hypertension	Prevention, diagnosis, follow-up	Effectiveness Efficiency Person-centredness

**Table 2.** Overview of grey literature retrieved, setting, models/frameworks used, and quality aims identified.

Author/editor/organisation (year)	Setting	Model(s)/framework(s)	Quality aims identified
World Health Organization (2013) [3]	Global	None	Effectiveness Efficiency Equity Person-centredness Continuity
World Health Organization (2016) [4]	Global	Integrated Care Models	Effectiveness Efficiency Person-centredness Continuity
World Health Organization, Organisation for Economic Co-operation and Development, and the World Bank (2018) [12]	Global	Elements of health care quality	Effectiveness Efficiency Equity Safety Person-centredness Timeliness Continuity (as aim of integration)
Institute of Medicine (US) Committee on Quality of Health Care in America (2001) [14]	USA, but also used worldwide	IOM Quality aims	Safety Effectiveness Patient-centredness Timeliness Efficiency Equity/equitability
Institute of Medicine (US) Committee on Quality of Health Care in America (2018) [15]	USA, but also used worldwide	IOM Quality aims	Effectiveness Efficiency Equity Safety Person-centredness Accessibility, timeliness, and affordability (Continuity)
Escobar et al. (2013) [67]	Pan-American Region, but also used worldwide	None	Effectiveness
World Health Organization (2008) [68]	Global	None	Effectiveness Efficiency Person-centredness Continuity
World Health Organization (2019) [69]	Global	None	Effectiveness Efficiency
World Health Organization (2022) [70]	Global	Health systems performance	Effectiveness Efficiency Equity Safety Person-centredness (as aim of user experience) Accessibility, timeliness, affordability
World Health Organization and the United Nations Children’s Fund (2018) [71]	Global	Primary Health Care	Effectiveness Person-centredness Continuity
World Health Organization (2014) [72]	Global	NCD assessment guide	Effectiveness
World Health Organization (2002) [73]	Global	None	Effectiveness Efficiency Person-centredness Continuity
Palmer et al. (2016) [74]	Europe	None	Effectiveness Person-centredness Continuity
EU Joint Action on Chronic Diseases and Healthy Ageing Across the Life Cycle (undated) [75]	Europe	practice quality evaluation tool	Effectiveness Person-centredness
EU Joint Action on Chronic Diseases and Healthy Ageing Across the Life Cycle (undated) [76]	Europe	QCR tool	Effectiveness Safety Person-centredness
EU Joint Action on Chronic Diseases and Healthy Ageing Across the Life Cycle (2019) [77]	Europe	QCR tool	Effectiveness Safety Person-centredness
Institute of Medicine (US) Committee on Quality of Health Care in America (1999) [78]	USA, but also used worldwide	None	Effectiveness Safety
Peikes, et al. (2014) [79]	USA	evaluation	Timeliness, accessibility Continuity
McDonald et al. (2014) [80]	USA	Care Coordination Measurement Framework	Effectiveness Safety Person-centredness Continuity
Thompson (undated) [81]	USA	Disease Management Program	Effectiveness Person-centredness Continuity
U.S Department of Health and Human Services (2010) [82]	USA	None	Effectiveness Person-centredness
Primary Health Care Advisory Group (2015) [83]	Australia	Health Care Home	Effectiveness Safety Person-centredness Accessibility Continuity
Paulus et al., eds (2012) [84]	Belgium	None	Effectiveness Person-centredness Continuity
Jackson et al. (2016) [85]	Canada	Continuity of Care	Continuity
Department of Health and Children (2008) [86]	Ireland	None	Effectiveness Patient/person-centredness Continuity
Quality Assurance Research and Policy Development Group (2004) [87]	Philippines	Quality Standards	Effectiveness Safety Person-centredness Accessibility, timeliness Continuity

More detailed information extracted from the scientific and grey literature can be found in the supplementary files, available from https://www.itg.be/en/research/research-themes/quality-of-care-for-chronic-conditions.

### Delphi survey

Forty-nine of the 52 invited stakeholders (94%) consented and participated in the Delphi survey. Table 3 provides demographic and pertinent characteristics.

**Table 3.** Demographic characteristics of Delphi respondents (*n* = 49).

Age	Average	49.1 years
	range	33–65 years
Sex	Female	15
	Male	34
Continent of origin	Africa	10
	Asia	12
	Europe	11
	North America	7
	Oceania	2
	South America	3
	Chose not to disclose	3
	No answer	1
Socio-economic classification of country/ies of ‘expertise’/having knowledge of	Low-income (LIC)	10
	Middle-income (MIC)	10
	High-income (HIC)	3
	All	7
	Both LIC and MIC	13
	Both MIC and HIC	3
	Not applicable	3
Stakeholder characteristics (multiple answers possible)	Clinician/health care provision	15
	Health financing	29
	Policy implementation	19
	Policy formulation	21
	Government adviser	33
	Teacher or researcher in chronic conditions	17
	Teacher or researcher in quality of care	24
	Teacher or researcher in health financing	20
	Informal caregiver of PwCC	5
	PwCC	4
	Civil society representative	2
	Healthcare organisation representative	8
	Patient group representative	4

### Specifying aims for good-quality chronic care

For quality of care for chronic conditions, we noted that the six aims as proposed by the IOM: effectiveness; efficiency; safety; equitability; accessibility, timeliness, and affordability; and person-centredness also apply. Additionally, we identified a seventh aim, continuity of care. There was consensus among the Delphi participants on our propositions; they also provided reasons why each proposed aim should be included. One panellist recommended giving enough attention to integration to organise achieving the aims.

*Effectiveness* is defined by the IOM [15] as the provision of services based on scientific knowledge to all who could benefit and refraining from providing services to those not likely to benefit (i.e. avoiding both overuse of inappropriate care and underuse of effective care). ‘Effectiveness’ was noted in 43 out of the 48 scientific articles [18–44,49–55,57–65] and in 24 of the 26 grey literature [3,4,12,14,15,67–78,80–84,86,87]. It is the most used quality aim, customarily measured with favourable (clinical) outcomes. Studies tend to utilise good clinical outcomes to demonstrate good quality of care or successful implementation of any model for chronic care. This is supported by various models of chronic care, where the ‘endpoints’ are good clinical (and functional) outcomes.

This outlook seems supported by the Delphi respondents:

*we need … services/interventions which are effective (yielding intended results)*

and that there is

*no point in continuing if (care is) ineffective*.

*Efficiency* is a quality aim in 20 of the scientific articles [26,28,30,32,39,43–49,51,52,54,55,57,61,62,65] and in 9 of the grey literature [3,4,12,14,15,68–70,72]. This aim is about using appropriate (amounts of) resources and avoiding waste, including waste of equipment, supplies, ideas, and energy [12]. Efficiency is effectuated, for instance, by reducing inappropriate use of emergency departments [28] and avoidable hospital admissions [32]; efficient use of time [57]; coordinating additional or specialised services only for PwCC who need these, etc. [12,85]. It also includes cost-efficient management that would lead to cost reductions without compromising beneficial effects [66], for instance the appropriate use of health technologies and information technology, coordinating additional or specialised services only for PwCC who need them, etc. [71].

A respondent highlighted efficiency and related this to affordability:

*Efficiency combines effectiveness at a more appropriate cost; this can already solve many access issues, namely financial access issues.*

*Safety* is defined by the IOM as avoiding harm to PwCC from the care that is intended to help them [14,78]. The WHO [12] specifies this as ‘patient safety’. However, the grey literature from the Philippines [87] gives a broader goal *to provide patients, staff, and other individuals within the health facility a safe environment*. This quality aim appeared in only three of the scientific literature we reviewed [29,39,52] and in 10 of the grey literature [12,14,15,70,76–78,80,83,87].

A respondent indicated that safety can be complex and affected by the context.

*… given the context in which the implementation takes place, with low or middle (income country) organizational conditions lacking material and sometimes motivation from the workforce, safety can be a problem, especially in remote areas…*

*Equity*, expressed as ensuring that all PwCCs can access good-quality healthcare that is responsive to their needs regardless of personal characteristics [12] were identified in four of the scientific articles [23,29,40,43] and four other grey literature [3,14,15,70].

A respondent indicated that:

*Inequity has always been a major challenge, not only due to socioeconomic classes, but also due to clientelism practiced by politicians, including health ministers, whereby ‘connected’ persons had better access to healthcare.*

*Accessibility, timeliness, and affordability*, defined as reducing unwanted waits and harmful delays for both those who receive and those who give care, reducing access barriers and financial risk for patients, families and communities and promoting affordable care for the system [15], are presented in full or as one/two of the component-aims in 20 of the scientific papers we have reviewed [21,29,31,38,39,42,43,45–47,49,50,52,56–61,64], and implied in two more [27,40]. The full aim is presented in two grey literature [15,70] and as one/two of the component-aims in four more [14,79,83,87].

Respondents spoke about geographic and financial accessibility and timeliness, and linked accessibility to equity.

*‘The biggest challenge is access to care for chronic diseases, such as diabetes, for all patients wherever they are, given the geographical complexity’. (*of specific countries)

*… there is a vast gap between the theoretical coverage of the social health insurance system and the actual coverage, which generates disparities in access to health care in general, particularly for people suffering from chronic conditions. This mainly affects people in rural areas and low-income communes in urban settings.*

*Patients should (be able to) access quality chronic care when they need, without any barrier.*

*… ensure that services of health providers are generally accessible to all and equitably distributed.*

*Person-centredness* is defined by WHO [70] as the approach to care that consciously adopts the perspectives of individuals, carers, families and communities as participants in, and beneficiaries of, trusted health systems organised around the comprehensive needs of people rather than individual diseases and respects social preferences. This is a central dimension in 36 of the scientific papers [18,20,29,31–39,41–44,48–53,58,62–65] and ranges from outright mentions of patient-/person-centredness to implicit indications that relate to collaborating with and engaging PwCC in their care and self-management. It is also included in 19 more grey literature [3,4,12,14,15,68,71,73–77,80–84,86,87] we reviewed.

In the Delphi, respondents connected person-centredness to access and talked about ‘empowerment’.

*Within a context of rapidly escalating co- and multi-morbidities, it is critical (to) reflect (on) mechanisms which create access to care for the ‘person’ and not the ‘disease or conditions’.*

*I would put a premium on empowering patients to be able to do self-care, particularly for interventions that have been proven to be effective.*

We identified a seventh aim, ‘continuity of care’, which is particularly important for people who require regular, consistent healthcare services for a long period of time. Continuity is among the recommendations by the Institute of Medicine in 2001 [14] (*Care based on continuous healing relationships*). The WHO’s ICCC Framework [73] extensively discusses continuity across time and care settings. *Continuity* is the focus of one of our grey literature (Jackson et al. [85]), where it is defined as the *degree to which a series of discrete health events is experienced as coherent and connected, and consistent with the patient’s healthcare needs and personal context*. It includes the capacity to monitor and respond to change, support self-management goals, and link to community resources [37]. It also includes follow-up and tracing of lost-to-follow-up [23,57]. Three sub-dimensions are given by Jackson et al. [85], which we utilised as a further basis for inductive and deductive analysis:

[i] Relational/relationship continuity, defined as *a trusting relationship with one or more HCP who help bridge healthcare episodes over time.*

[ii] Informational continuity, where health information as well as other relevant information about the PwCC (their values, preferences, contexts) are shared.

(i.e., shared medical records)

[iii] Management continuity, where patient-related information regarding their case management is communicated to different relevant HCP.

Continuity is mentioned in 14 other grey literature [3,4,12,68,71,73,74,79–81,83,84,86,87]. It is also explicitly included as a quality dimension in five of the scientific literature we retained [23,29,37,40,49] and implied in 17 more [19–21,28,31,33,37,39,41,42,51,52,56,57,59,61,63].

Respondents placed value on continuity as a quality aim, especially for chronic care.

*… continuity of care is probably the major issue regarding chronic diseases …*

*A lack of continuity of care is currently one of the main reasons patients who access the system fall through the cracks.*

*Continuum of care is vital for NCDs…*

Based on our definition, ‘*Integration*’ does not qualify as an aim. We note that one grey literature refers to it as a measurable characteristic of quality of care (i.e. considered as an aim) [12] and another as a necessary characteristic of (people-centred) health care [84]. One grey literature focuses on integration, where it is described as an action or a process [4]. Thirteen scientific literature [24,39–41,43,44,46,49,51,61–63,65] we reviewed mention it as instrumental to improving quality of care for chronic conditions.

One of the Delphi respondents indicated:

*a key thing is the principle (the ‘what’), and that is the principle of integrated chronic care. Right now, we have reactive, short-term care … changing that mindset is priority*.

Another stated that

*for improved care on chronic conditions we need to achieve … delivery within integrated care pathways spanning across (the) care sector and being organised around the patient …*

## Discussion

Chronic conditions raise particular issues in healthcare because they last for a long time and, more often, throughout the lifetime of the person. Another consideration is the natural history of chronic conditions and the ‘journey’ of a PwCC through time, traversing the natural history. To respond to these, healthcare services should encompass risk- and disease-prevention, clinical management of the condition and any complications and/or multi-morbidity, rehabilitation and/or community re-integration, palliative/end-of-life care, and considering the psychosocial aspects of the PwCC. Additionally, clinical/biomedical control of chronic conditions is not static. PwCCs experience episodes of good and poor control of the condition at different moments throughout their lifetime. Control of the condition can be affected by many factors such as continued exposure to risks and determinants, other health problems, e.g. infections, psychological issues (anxiety, depression, etc.), co-morbidity, suboptimal clinical management by either the healthcare system or by the PwCC/informal caregivers themselves, as well as social factors (e.g. lack of social support). These redound to specific healthcare needs and corresponding services and support the view to reflect on how the quality of such healthcare would be defined.

We have formulated a definition and determined quality aims paying attention to the specificities of chronic conditions and their care. We define quality of chronic care *as the degree to which healthcare services for individuals and populations with chronic conditions – including provision of education and support to adapt and self-manage in the face of social, physical, and emotional challenges – which are consistent with current professional knowledge and increase the likelihood of desired health outcomes and biopsychosocial well-being*. This complements our chronic care quality aims, wherein achievement of each is influenced by and would contribute to realisation of our definition.

Regarding the aims, we noted from the literature review and the Delphi survey that most of the IOM care quality aims – namely effectiveness, safety, equity, person-centredness, and accessibility – take on new, additional meanings specific for chronic care.

Further to ‘effectiveness’ as a chronic care quality aim is the consideration that people develop chronic condition(s) due to exposure to various risk factors and social determinants of health [18,20]. These would also still affect the person even if they already have a chronic condition, and increase the propensity of having poor clinical control, emergence of complications and/or development of other morbidities [30]. Thus, effective health services to prevent and control risks, e.g. healthy lifestyle promotion, smoking cessation counselling, etc., should be provided to the general population and the PwCCs (in addition to their effective case management). This was also pointed out by our Delphi survey respondents. Logically, failure to provide effective services to prevent and control risks would increase the number of PwCCs and could give rise to multi-morbidity. Compounding this with failure to deliver effective chronic care would further increase the burden of chronic conditions. However, an understanding of the responsibilities of the health system needs to be clarified. Addressing the various risks and social determinants themselves, e.g. air pollution control, increasing access to healthy food, regulation of sales of unhealthy products, food (re)formulations, tobacco and alcohol taxations, etc., would need actions beyond the scope of the health system, even if the health system may initiate multi-sectoral policies and actions [88]. Furthermore, effectiveness should not be limited to chronic care provision alone. PwCC are often immunocompromised, making them susceptible to various infections. Responsive health systems should thus have the capacity and capability to effectively provide care for both chronic conditions and acute infections and support the psychosocial needs of PwCC.

With the ballooning burden of chronic conditions and higher demands for chronic care, efficiency becomes a dire necessity across all settings. Whilst efficiency can be looked at individual level (best use of own resources), it has more relevance as an aim at collective level. From the societal perspective, efficiency ensures that what is not wasted on one person is available for the other who truly needs it. Efficiency, especially of chronic care, is one of the main aims of many recent health system reforms, which are, however, being implemented mainly in high-income countries.

We echo one of our Delphi respondents that safety is indeed complex and largely depends on the context. Moving from concentrating only on patient safety, we favour the more extensive safety aim presented in the PhilHealth Bench Book [87], which includes measures for both patients and (healthcare) staff and providing a safe environment for healthcare delivery. In addition, effects of chronic care delivery on the environment should also be considered; for instance, cytostatic drugs excreted by cancer patients finding their way to wastewater and affecting biodiversity of freshwater organisms. Concrete examples of broader safety include: having appropriate facility design, e.g. to accommodate people with disabilities; prevention of adverse care incidents, e.g. drug-induced hypoglycaemia, drug–drug interactions in cases of polypharmacy (especially among PwCC with comorbidities/multi-morbidity); provision of equipment and devices needed to deliver safe care, e.g. personal protective equipment; proper waste handling, including proper disposal of sharps used by both the staff and PwCC, appropriate wastewater treatment, etc. While safety was the least studied quality aim among the papers we reviewed, its importance in chronic care and its wider application including effects on global environmental changes cannot and should not be discounted.

Equity is a cross-cutting consideration in our societies. It can be assessed over any metric of interest and consists of a normative judgement on the distribution within a group of persons; it is usually not an aim at individual level, but at collective level. For instance, there can be equity considerations on how households financially contribute to the general funding of the health system, with different views on what would be fair. Health systems performance and UHC literature has put equity as a core consideration [70]. Akin to equity in healthcare in general and as defined by the IOM, we propose to refer to equity as the aim capturing distributional considerations related to quality of chronic care. The first concern should be that care does not vary in quality (effectiveness, safety, timeliness, person-centredness, etc.) because of personal characteristics such as gender [64], ethnicity, socioeconomic status, disabilities, etc. [29]. Such fairness redounds in health promotion and all levels of prevention of chronic conditions, especially in LMICs where exposure to risks and social determinants, and the prevalence of most chronic conditions have been documented to be higher among lower socio-economic groups, and where more inequities have also been noted (e.g. between sexes, ethnicity).

As regards individual PwCC, we deduce, and view, person-centredness to be more holistic and more all-encompassing than what is defined by both IOM and WHO. To deliver person-centred chronic care, what should be strived for should go beyond the ‘*patient is a person*’ concept. The recognition and acceptance should be framed as follows: the ‘*person is sometimes a patient*’ and the ‘*patient is always a person*’. The biomedical, psychological and social aspects of PwCC need to be considered [10,12,50,52]. They should be ‘activated’/stimulated to become experts of their condition and should be guided to accept and recognise that their chronic condition is only one of the facets of their whole life. Emphasis is given on their *human agency* and their capacity to make their own decisions, set goals, take actions, and sustain efforts. Therefore, beyond empowerment/enablement for self-management, there should be engagement of the PwCC for collaborative care and involvement in applicable committees to improve chronic care quality [25,42,71]. Such engagement extends to their family, their informal caregivers and the community [23,39,49,63]. A goal would be that PwCCs will be able to ‘juggle’ the different facets of their life effectively to maximise their physical/biological, psychological and social well-being, and are supported accordingly.

Accessibility, affordability, and timeliness are mainly instrumental to other aims. However, they also have intrinsic value. Following Levesque et al.’s [89] proposition, we further dissect this aim as applied to chronic care:

- Geographic accessibility – to help enable PwCC to consult and follow-up regularly without having to travel a great distance and/or encountering too many difficulties in travelling and/or losing much time for travel and/or incurring unreasonable transportation expenses [29,61]. It is a source of reassurance: for PwCCs to know that in case of need, there is a solution nearby. Less uncertainty reduces stress; it also expands choices for daily life.
- Financial accessibility (*affordability)* of services, medications and supplies – as too-high and/or recurring financial costs charged to the PwCCs compromise their welfare and may force PwCC to stop their maintenance medications (e.g. cannot buy high-priced insulin) or cause *iatrogenic poverty* (e.g. need to go into debt to finance chemotherapy) [29,47,49,52]. It also frees resources for other needs.
- Temporal accessibility – with considerate opening hours, and reasonable waiting times (e.g. for consultations) and turn-around-times (e.g. of laboratory results) [21,38,39,47] as long waiting times for laboratory test results or to receive appropriate care can create distress and anxiety, if not the worsening of the condition.
- Availability of chronic care services, including diagnostics and when, i.e. periodic or consistent, and availability of medications [27,31,42,43,46,49,50,56,60,64] – considering that making services and diagnostics available either every day or at regular specific schedules would be optimal for a PwCC who will need to utilise these services repeatedly; as well as
- Health care worker-related factors, e.g. cultural congruence, approachability [36,46,49,63,64] – as these highly contribute to acceptability, prompting better access, and building trust.

The effects of achieving the different sub-dimensions of the above aim are many-fold: improving chronic care delivery and utilisation of healthcare services, contributing to improved adherence of PwCC, improving (clinical) outcomes, etc. Achieving this aim would directly contribute to the achievement of good-quality chronic care, as we defined.

The intrinsic value of our seventh aim, continuity, is how it acknowledges that time and its continuity (past, present and future) matter for the PwCC. It addresses chronicity, which can be discerned from the three subdimensions proposed by Jackson et al. [85].

[i] Relational/relationship continuity, as establishing rapport and trust between the HCP and the PwCC would be crucial to build a lasting relationship, help ensure regular follow-up, and more likely promote (long-term) adherence [29,39,51,52].

[ii] Informational continuity, to ensure timely availability of the health information of PwCCs across different HCP as they navigate different healthcare disciplines, different levels of care, through time, including any changes of HCP (e.g. when PwCC relocate) [19–21,28,29,31,33,37,39,41,42,49,56,63]; and

[iii] Management continuity, where patient-related information regarding their case management is communicated to relevant HCP and shared with the PwCC themselves and/or their informal caregivers. This way, care is delivered across different health sectors and by multiple HCP as well as by the PwCC themselves and/or their informal caregivers in a coherent, logical and timely fashion [19,21,28,31,33,37,39,41,42,49,56,61,63].

Informational and management continuity go hand-in-hand, where relevant information about the PwCC (informational continuity) as well as care management plans (management continuity) would be co-developed and shared with relevant care providers (including informal caregivers) and the PwCC and would follow the PwCC in their journey through healthcare.

A thorny issue is the status of ‘integration’, for which multiple definitions have been given [4]. Certainly, poor and insufficient integration of service delivery is a major issue in many countries given the current state of their health systems. This observation, shared across settings including in high-income countries, probably motivated the decision to elevate integration as an aim in another quality of care framework [12]. However, we propose to distinguish the conceptual undertaking of identifying the aims of quality of care from policy agendas inspired by current situations. From a conceptual perspective, our assessment is that integration does not meet the intrinsic value criterion of an aim nor is it a directional metric (for which any progress is valuable). It, however, plays a special role in care quality. Good-quality chronic care requires integration. However, integration is not the goal per se (in contrast to the seven dimensions of quality of chronic care), but is a means: it is instrumental in achieving quality aims. The process of integration or the action of integrating healthcare organisations and/or its people and/or its services and/or its functions – to deliver integrated care – can help achieve, more particularly, effectiveness, efficiency, person-centeredness, and continuity. To a lesser extent, it can also contribute towards accessibility and safety. Conceptually, integration is desirable, but only to the extent that it positively serves quality aims. Empirically, we acknowledge that it should be a top priority in all health systems; a way to organise actions to improve quality of (chronic) care. Our proposition to elevate integration as an organising principle to achieve the aims of quality chronic care will be discussed in a separate paper.

## Limitations

Available literature on quality of (chronic) care mostly documents experiences in high-income countries, this was also the case in our scoping review. We mitigated this limitation by purposively selecting Delphi participants with expertise and experiences on chronic care in LMICs.

We also noted that specific aims got more attention over others (for instance, effectiveness was the focus of almost all of the scientific literature we reviewed, while less than 10 were on equity, safety). While this may be an unintended effect of our literature search, we noted that effectiveness is usually the *de facto* aim that is studied because of the value placed on desired clinical outcomes as the endpoint. However, this does not warrant inattention to the other aims. The inclusion of grey literature, most of which tackled all aims, made sure that we have a broad understanding even of the least studied aims in the scientific literature.

We have adopted the view that ‘aims’ should be intrinsically valuable. It is important to understand that this does not mean that non-included aims are irrelevant.

Although we introduced the PwCC journey and the relevance of the various stages in a continuum of care relevant to the identified aims, we are aware that we did not have room to expound more on this concept. We will discuss this in more detail in a separate paper presenting our conceptual quality of chronic care framework.

## Conclusions

With this paper, we have moved from a generic understanding of quality of care to one tailored to chronic conditions. Beyond aims, we have also determined the scope of attention, one which values a comprehensive offer of healthcare services, addresses risks and social determinants of health, ensures biopsychosocial well-being of PwCC, and gives importance to quality of care characteristics relevant to the PwCC and their families, to the community, and to the health system.

Our scoping review shows that some aims have received more attention than others. However, limited attention should not be interpreted as an acceptable reason for neglect. Our Delphi survey respondents underlined the value of each of the seven chronic care quality aims that we identified, especially as applied to low-income settings.

## Implications for policy & further research

Our team used these aims to create a chronic care quality framework, and then, via the Delphi survey, mobilised the international panel of experts to apply the said framework for possible purchasing arrangements to improve quality of chronic care in LMICs. The chronic care quality framework and the Delphi survey results on purchasing arrangements will be presented separately. These are all components of the larger programme of work implemented by WHO, which focuses on purchasing arrangements as an instrument to improve health services for chronic conditions. It is expected that member states will take inspiration from this programme of work, in their efforts to improve care for chronic conditions. Actors active in chronic care may also be inspired by our specifications, in designing good-quality chronic care services or working on improvement strategies thereto.

The output we presented in this paper is conceptual. Operationalisation for systematic improvements in quality of chronic care can be the next step, among others, to demonstrate the usefulness, or not, of each of these specified chronic care quality aims, in specific settings.

Our paper may also inspire other calibrations and validation of the definition and aims of quality of care for other health problems. We hypothesise that they could be valuable preparatory steps among those committed to improve quality of care for specific health conditions. Having a tailored understanding of quality of care will only make quality improvement interventions better.

## Pre-print

A copy of the original submission is available fromhttps://www.medrxiv.org/content/10.1101/2024.04.05.2430

## References

[1] National Center for Chronic Disease Prevention and Health Promotion. About Chronic Diseases [Internet]. USA: US Department of Health and Human Services; Undated [last revised 2022, cited July 30 2021. Sep]. Available from 21. Available from: https://www.cdc.gov/chronicdisease/about/index.htm

[2] World Health Organization. Integrated management of adolescent and adult illness (IMAI): interim guidelines for first level health facility health workers. Geneva: WHO; 2004.

[3] World Health Organization. Global action plan for the prevention and control of non-communicable diseases 2013-2020. Geneva: WHO; 2013.

[4] World Health Organization Europe. Integrated care models: an overview. Copenhagen: WHO Regional Office for Europe; 2016.

[5] Busse R, Klazinga N, Panteli D & Quentin W, editors. Improving healthcare quality in Europe: characteristics, effectiveness and implementation of different strategies. UK: European Observatory on Health Systems and Policies & WHO; 2019. 31721544

[6] Funnel MM. Helping patients take charge of their chronic illnesses. Fam Pract Manag. 2000;7:47–19. 10947289

[7] Longino CF, Pardeck JT, Murphy JW. Reason and rationality in health service delivery. (NY): Haworth Press; 1998. 10.1300/J045v09n04_01 10180596

[8] World Health Organization. The Ljubljana charter on reforming health care. BMJ. 1996;312:1664–1665. doi: 10.1136/bmj.312.7047.1664 8664727 PMC2351397

[9] De Ridder D, Geenen R, Kuijer R, van Middendoerp H. Psychological adjustments to chronic disease. The Lancet. 2008;372:246–255. doi: 10.1016/S0140-6736(08)61078-8 18640461

[10] Huber M, Knottnerus JA, Green L, Horst H VD, Jadad AR, Kromhout D, et al. How should we define health? BMJ. 2011;343:d4163. doi: 10.1136/bmj.d4163 21791490

[11] Schmittdiel JA. Effect of primary health care orientation on chronic care management. Ann Med. 2006;4:117–123. doi: 10.1370/afm.520 PMC1467016 16569714

[12] World Health Organization. Organisation for economic cooperation and development, and the World Bank. Delivering quality health services: a global imperative for universal health coverage. Geneva: WHO, OECD and The World Bank; 2018.

[13] Lohr KN. Medicare: a strategy for quality assurance. J Qual Assur. 1991 Jan-Feb;13:10–13. doi: 10.1111/j.1945-1474.1991.tb00115.x PMID: 10109548. 10109548

[14] Institute of Medicine (US). Committee on quality of health care in America. Crossing the quality chasm: a new health system for the 21st Century. Washington, D.C: National Academies Press; 2001. 25057539

[15] National Academies of Sciences, Engineering, and Medicine. Crossing the global quality chasm: improving health care worldwide. Washington (DC): National Academies Press; 2018. 30605296

[16] Tricco AC, Lillie E, Zarin W, O’brien KK, Colquhoun H, Levac D, et al. PRISMA extension for scoping reviews (PRISMA-ScR): checklist and explanation. Ann Intern Med. 2018;169:467–473. doi: 10.7326/M18-0850 30178033

[17] Vos T, Lim SS, Abbafati C. GBD. 2019 diseases and injuries collaborators. Global burden of 369 diseases and injuries in 204 countries and territories, 1990–2019: a systematic analysis for the global burden of disease study 2019. The Lancet. 2020;396:1204–1222. doi: 10.1016/S0140-6736(20)30925-9 PMC7567026 33069326

[18] Hung DY, Rundall TG, Tallia AF, Cohen DJ, Halpin HA, Crabtree BF. Rethinking prevention in primary care: applying the chronic care model to address health risk behaviors. Milbank Q. 2007;85:69–91. doi: 10.1111/j.1468-0009.2007.00477.x 17319807 PMC2690311

[19] Lewanczuk R. Innovations in primary care: implications for hypertension detection and treatment. Can J Cardiol. 2006;22:614–616. doi: 10.1016/S0828-282X(06)70284-7 16755317 PMC2560870

[20] Hung DY, Glasgow RE, Dickinson LM, Froshaug DB, Fernald DH, Balasubramanian BA, et al. The chronic care Model and relationships to patient health status and health-related quality of life. Am J Prev Med. 2008;35:S398–S406. doi: 10.1016/j.amepre.2008.08.009 18929987

[21] Hroscikosky MC, Solberg AI, Sperl-Hillen JM, Harper PG, Mp M, Crabtree BF. Challenges of change: a qualitative study of chronic care Model implementation. Ann Fam Med. 2006;4:317–326. doi: 10.1370/afm.570 16868235 PMC1522157

[22] Jansen DL, Heijmans M, Rijken M. Individual care plans for chronically ill patients within primary care in the Netherlands: dissemination and associations with patient characteristics and patient-perceived quality of care. Scand J Prim Health Care. 2015;33:100–106. doi: 10.3109/02813432.2015.1030167 25961964 PMC4834496

[23] Kaissi AA, Parchmann M. Assessing chronic illness care for diabetes in primary care clinics. Joint Commission J On Qual Patient Saf. 2006;32:318–323. doi: 10.1016/S1553-7250(06)32041-7 16776386

[24] Lim LL, Lau ES, Kong AP, Davies MJ, Levitt NS, Eliasson B, et al. Aspects of multicomponent integrated care promote sustained improvement in surrogate clinical outcomes: a systematic review and meta-analysis. Diabetes Care. 2018;41:1312–1320. doi: 10.2337/dc17-2010 29784698 PMC5961399

[25] Ludt S, Van Lieshout J, Campbell SM, Rochon J, Ose D, Freund T, et al. Identifying factors associated with experiences of coronary heart disease patients receiving structured chronic care and counselling in European primary care. BMC Health Serv Res. 2012;12:12. doi: 10.1186/1472-6963-12-221 22838403 PMC3660215

[26] Lyon RK, Slawson JG. An organized approach to chronic disease care. Fam Pract Manag May–June. 2011;18 1:27–31. 21842806

[27] Vrijhoef HJM, Berbee R, Wagner EH, Steuten LMG. Quality of integrated chronic care measured by patient survey: identification, selection and application of most appropriate instruments. Health Expectations. 2009;12:417–429. doi: 10.1111/j.1369-7625.2009.00557.x 19709315 PMC5060503

[28] Petrelli F, Cangelosi G, Nittari G, Pantanetti P, Debernardi G, Scuri S. Chronic care Model in Italy: a narrative review of the literature. Prim Health Care Res Dev. 2021;22:1–7. doi: 10.1017/S1463423621000268

[29] Lall D, Engel N, Devadasan N, Horstman K, Criel B. Models of care for chronic conditions in low/middle-income countries: a ‘best fit’ framework synthesis. BMJ Glob Health. 2018;3:e001077. doi: 10.1136/bmjgh-2018-001077 PMC6326308 30687524

[30] Parchman N, Kaissi AA. Are elements of the chronic care model associated with cardiovascular risk factor control in type 2 diabetes? Joint Commission J On Qual & Patient Saf. 2009 3;35:135–138. doi: 10.1016/S1553-7250(09)35017-5 19326804

[31] Mateo-Gavira I, Carrasco-García S, Larran L, Fierro MJ, Zarallo A, Mayoral Sánchez E, et al. Specific model for the coordination of primary and hospital care for patients with diabetes mellitus. Evaluation of two-year results (2015–2017). Endocrinología, Diabetes y Nutrición (Engl ed). 2021;68:175–183. doi: 10.1016/j.endien.2021.05.002 34167697

[32] Enderlin CA, McLeskey N, Rooker JL, Steinhauser C, D’Avolio D, Gusewelle R, et al. Review of current conceptual models and frameworks to guide transitions of care in older adults. Geriatric Nurs. 2013;34:47–52. doi: 10.1016/j.gerinurse.2012.08.003 23122908

[33] Sendall M, McCosker L, Crossley K, Bonner A. A structured review of chronic care model components supporting transition between healthcare service delivery types for older people with multiple chronic diseases. Health Inf Manag J. 2016;46:58–68. doi: 10.1177/1833358316681687 27923916

[34] Hopman P, De Bruin SR, Forjaz MJ, Rodriguez-Blazquez C, Tonnara G, Lemmens LC, et al. Effectiveness of comprehensive care programs for patients with multiple chronic conditions or frailty: a systematic literature review. Health Policy. 2016;120:818–832. doi: 10.1016/j.healthpol.2016.04.002 27114104

[35] Adams JS, Woods ER. Redesign of chronic illness care in children and adolescents: evidence for the chronic care model. Curr Opin Pediatr. 2016;28:428–433. doi: 10.1097/MOP.0000000000000368 27138998

[36] Litzelmann K. Caregiver well-being and the quality of cancer care. Semin Oncol Nurs. 2019;35:348–353. doi: 10.1016/j.soncn.2019.06.006 31229346 PMC6728914

[37] Dugoff EH, Dy S, Giovannetti ER, Leff B, Boyd CM. Setting standards at the forefront of delivery system Reform: aligning care coordination quality measures for multiple chronic conditions. J Healthc Qual. 2013;35:58–69. doi: 10.1111/jhq.12029 24004040 PMC4446982

[38] Brand CA, Ackerman IN, Tropea J. Chronic disease management: improving care for people with osteoarthritis. Best Pract Res Clin Rheumatol. 2014;28:119–142. doi: 10.1016/j.berh.2014.01.011 24792948

[39] Buja A, Toffanin R, Claus M, Ricciardi W, Damiani G, Baldo V, et al. Developing a new clinical governance framework for chronic diseases in primary care: an umbrella review. BMJ Open. 2018;8:e020626. doi: 10.1136/bmjopen-2017-020626 PMC6067352 30056378

[40] Beland F, Hollander MJ. Integrated models of care delivery for the frail elderly: international perspectives. Gac Sanit. 2011;25:138–146. doi: 10.1016/j.gaceta.2011.09.003 22088903

[41] Kanter MH, Lindsay G, Bellows J. Complete care at Kaiser Permanente: transforming chronic and preventive care. Joint Commission J On Qual Patient Saf. 2013;39:484–494. doi: 10.1016/S1553-7250(13)39064-3 24294676

[42] Chiu HHL, Murphy-Burke DMM, Thomas SA, Yuriy M, Kruthaup-Harper AL, Janghu JD, et al. And the BC renal palliative committee. Advancing palliative care in patients with CKD: from ideas to practice. Am J Kidney Dis. 2020;77:420–426. doi: 10.1053/j.ajkd.2020.09.012 33181264

[43] Morrin L, Britten J, Davachi S, Knight H. Alberta healthy living program–A Model for successful integration of chronic disease management services. Can J Diabetes. 2013;37:254–259. doi: 10.1016/j.jcjd.2013.04.001 24070890

[44] Nuno R, Coleman K, Bengoac R, Sautoa R. Integrated care for chronic conditions: the contribution of the ICCC framework. Health Policy. 2012;105:55–64. doi: 10.1016/j.healthpol.2011.10.006 22071454

[45] Lebina L, Oni T, Alaba OA, Kawonga M. A mixed methods approach to exploring the moderating factors of implementation fidelity of the integrated chronic disease management model in South Africa. BMC Health Serv Res. 2020;20:617. doi: 10.1186/s12913-020-05455-4 32631397 PMC7336628

[46] Ameh S, Fx G-O, Kahn K, Tollman SM, Klipstein-Grobusch K. Relationships between structure, process and outcome to assess quality of integrated chronic disease management in a rural South African setting: applying a structural equation model. BMC Health Serv Res. 2017;17:229. doi: 10.1186/s12913-017-2177-4 28330486 PMC5363044

[47] Ameh S, Klipstein-Grobusch K, D’ambruoso L, Kahn K, Tollman SM, Gomez-Olive FX. Quality of integrated chronic disease care in rural South Africa: user and provider perspectives. Health Policy Plan. 2017;32:257. doi: 10.1093/heapol/czw118 28207046 PMC5400067

[48] Ulbrich EM, Mattei AT, MdF M, Bittencourt Madureira A, Puchalski Kalinke L. Care models for people with chronic diseases: integrative review. Invest Educ Enferm. 2017;35:8–16. doi: 10.17533/udea.iee.v35n1a02 29767919

[49] Grover A, Joshi A. An overview of chronic disease models: a systematic literature. Global J Health Sci. 2015;7:210–227. doi: 10.5539/gjhs.v7n2p210 PMC4796376 25716407

[50] Disler RT, Currow DC, Phillips JL, Smith T, Johnson MJ, Davidson PM. Interventions to support a palliative care approach in patients with chronic obstructive pulmonary disease: an integrative review. Int J Nurs Stud. 2012;49:1443–1458. doi: 10.1016/j.ijnurstu.2012.02.004 22405402

[51] Kari H, N A0-J, Kortejarvi H. Effectiveness and cost-effectiveness of a people-centred care model for community-living older people versus usual care ─ a randomised controlled trial. Res In Soc And Administrative Pharm. 2021;18:3004–3012. doi: 10.1016/j.sapharm.2021.07.025 34344607

[52] Kamajian S. Utilizing medical homes to manage chronic conditions. Osteopathic Fam Physician. 2010;2:102–107. doi: 10.1016/j.osfp.2010.01.002

[53] Brownson CA, Miller D, Crespo R, Neuner S, Thompson J, Wall JC. A quality improvement tool to assess self-management support in primary care. The Joint Commission J On Qual And Patient Saf. 2007;33:408–416. doi: 10.1016/S1553-7250(07)33047-X 17711143

[54] Harvey G, Oliver K, Humphreys J, Rothwell K, Hegarty J. Improving the identification and management of chronic kidney disease in primary care: lessons from a staged improvement collaborative. Int J For Qual In Health Care. 2015;27:10–16. doi: 10.1093/intqhc/mzu097 PMC4340270 25525148

[55] Hayashino Y, Suzuki H, Yamazaki K, Goto A, Izumi K, Noda M. A cluster randomized trial on the effect of a multifaceted intervention improved the technical quality of diabetes care by primary care physicians: the Japan diabetes outcome intervention trial-2 (J- DOIT 2). Diabet Med. 2015;33:599–608. doi: 10.1111/dme.12949 26331280 PMC5057414

[56] Hirschhorn LR, Landers S, Mcinnes DK, Malitz F, Ding L, Joyce R, et al. Reported care quality in federal Ryan White HIV/AIDS program supported networks of HIV/AIDS care. AIDS Care. 2009;21:799–807. doi: 10.1080/09540120802511992 19484615

[57] Joseph JP, Jeromea G, Lamberta W, Almazor P, Cupidon CE, Hirschhorn LR. Going beyond the vertical: leveraging a national HIV quality improvement programme to address other health priorities in Haiti. AIDS. 2015;29:S165–S173. doi: 10.1097/QAD.0000000000000715 26102627

[58] Pullen R, Miravitlles M, Sharma A, Singh D, Martinez F, Hurst JR, et al. CONQUEST quality standards: for the collaboration on quality improvement initiative for achieving excellence in standards of COPD care. Int J Chron Obstruct Pulmon Dis. 2021;16:2301–2322. doi: 10.2147/COPD.S313498 34413639 PMC8370848

[59] Wellwood I, Wu O, Langhorne P. Developing a tool to assess quality of stroke care across European populations: the EROS quality assessment tool. Stroke. 2011;42:1207–1211. doi: 10.1161/STROKEAHA.110.598938 21474805

[60] Hawthorne G, Hrisos S, Stamp E, Baradaran HR. Diabetes care provision in UK primary care practices. PLOS ONE. 2012;7:e41562. doi: 10.1371/journal.pone.0041562 22859997 PMC3408463

[61] Fletcher GF, Berra K, Fletcher BJ, Gilstrap L, Wood MJ. The integrated team approach to the care of the patient with cardiovascular disease. Curr Probl Cardiol. 2012;37:369–397. doi: 10.1016/j.cpcardiol.2012.04.001 22884247

[62] Mitchell JD, Haag JD, Klavetter E, Beldo R, Shah ND, Baumbach LJ, et al. Development and implementation of a team-based, primary care delivery Model: challenges and opportunities. In: Mayo Clinic Proceedings; 2019;94. p. 1298–1303. 10.1016/j.mayocp.2019.01.038 31272572

[63] Van Houtven CH, Hastings SN, Colón-Emeric C. A path to high-quality team-based Care for people with serious illness. Health Aff. 2019 6;38:934–940. doi: 10.1377/hlthaff.2018.05486 31158020

[64] Washington DL, Bean-Mayberry B, Mitchell MN, Riopelle D, Yano EM. Tailoring VA primary care to women veterans: association with patient-rated quality and satisfaction. Women’s Health Issues. 2011;21–4:S112–S119. doi: 10.1016/j.whi.2011.04.004 21724130

[65] Campbell N, Young ER, Drouin D, Legowski B, Adams MA, Farrell J, et al. A framework for discussion on how to improve prevention, management, and control of hypertension in Canada. Can J Cardiol. 2012;28:262–269. doi: 10.1016/j.cjca.2011.11.008 22284588

[66] Donabedian A. An introduction to quality assurance in healthcare. (NY): Oxford University Press; 2003.

[67] Escobar MC, Williams N, Alves AJ, Barcelo A, Prieto E, Delon S, Prieto F, Brebnor F. Manual for implementing quality care for chronic conditions. Brazil: WHO PAHO, Chronic Illness Care, CARMEN; 2022]. Available from Oct Jan Sep Available from [Available from], https://apsredes.org/wp-content/uploads/sites/2/2013/10/MQCCC.pdf [Available from Available from 3: 25 7].

[68] World Health Organization. Primary care: putting people first. In: Evans T and Van Lerberghe W (editor-in-chief). eds.The world health report 2008 “primary health care: now more than ever. Geneva: WHO; 2008. p. 41–60.

[69] World Health Organization. Assessing national capacity for the prevention and control of non-communicable diseases. Geneva: WHO; 2019.

[70] World Health Organization. Health system performance assessment. Geneva: WHO; 2022.

[71] World Health Organization and the United Nations Children’s Fund. A vision for primary care in the 21st century: towards universal health coverage and the sustainable development goals. Geneva: WHO and UNICEF; 2018.

[72] World Health Organization. Better NCD outcomes: challenges and opportunities for health systems. Geneva: WHO; 2014.

[73] World Health Organization. Innovative care for chronic conditions. Geneva: WHO; 2002.

[74] Palmer and the EU Joint Action on Chronic Diseases and Healthy Ageing Across the Life Cycle. Multimorbidity care model: recommendations from the consensus meeting of the joint action on chronic. Brussels: EC; 2016. 10.1016/j.healthpol.2017.09.006 28967492

[75] EU Joint Action on Chronic Diseases and Healthy Ageing Across the Life Cycle, undated. QCR tool based on CHRODIS: recommendations to improve prevention and quality of care for people with chronic diseases. Brussels: EC.

[76] EU Joint Action on Chronic Diseases and Healthy Ageing Across the Life Cycle, undated. CHRODIS QCR toolguide. Brussels: EC.

[77] EU Joint Action on Chronic Diseases and Healthy Ageing Across the Life Cycle. Deliverable 7.1 WP7 Pilot action design: a blueprint for action. Brussels: EC; 2019.

[78] Institute of Medicine (US). Committee on quality of health care in America. To err is human: building a safer health system. (WA) (DC): National Academies Press; 1999. 25077248

[79] Peikes D, Taylor EF, Genevro J, Meyers D. A Guide to real-world evaluations of primary care interventions. USA: Agency for Health Care Research & Quality; 2014.

[80] McDonald KM. Care coordination atlas. USA: Agency for Health Care Research & Quality; 2014.

[81] Thompson L. Disease management programs: improving health while reducing costs? In: The Center on an Aging Society at Georgetown University’s Institute for Health Care Research and Policy. Issue briefs on challenges for the 21st century: chronic and disabling conditions. Washinton (DC): George University Health Policy Institute; undated [cited 2022 Sep 7]. Available from: https://hpi.georgetown.edu/management/

[82] U.S. Department of Health and Human Services. Multiple chronic conditions - a strategic framework. Washington (DC): U.S. Department of Health and Human Services; 2010.

[83] Primary Health Care Advisory Group. Better outcomes for people with chronic and complex conditions. Australia: Department of Health (Australia); 2015.

[84] Paulus D, Van Den Heede K, Mertens R, editors. Organisation of care for chronic patients in Belgium: development of a position paper. Health services research (HSR). Brussels: Belgian Health Care Knowledge Centre (KCE); 2012.

[85] Jackson J, Lahtinen M, Cooke T. Understanding patient and provider experience with relationship, management and informational continuity. Canada: Health Quality Council of Alberta; 2016.

[86] Department of Health & Children. Tackling chronic disease: a policy framework for the management of chronic diseases. Ireland: Department of Health & Children; 2008.

[87] Philippine Health Insurance Corporation. Bench book on performance improvement of health services. Philippines: PhilHealth; 2004.

[88] Shroff ZC, Marten R, Hanson K, editors. Systems for health: everyone has a role. Flagship report of the alliance for health policy and systems research. Geneva: World Health Organization; 2022.

[89] Levesque JF, Harris MF, Russel G. Patient-centred access to health care: conceptualising access at the interface of health systems and populations. Intern J Equity In Health. 2013;12:18. doi: 10.1186/1475-9276-12-18 PMC3610159 23496984

